# Artificial intelligence as diagnostic modality for keratoconus: A systematic review and meta-analysis

**DOI:** 10.1016/j.jtumed.2023.12.007

**Published:** 2024-01-01

**Authors:** Azzahra Afifah, Fara Syafira, Putri Mahirah Afladhanti, Dini Dharmawidiarini

**Affiliations:** a Undaan Eye Hospital, Surabaya, Indonesia; b Medical Profession Program, Faculty of Medicine, Universitas Sriwijaya, Palembang, South Sumatra, Indonesia; c Lens, Cornea and Refractive Surgery Division, Undaan Eye Hospital, Surabaya, Indonesia

**Keywords:** الذكاء الاصطناعي, طريقة التشخيص, القرنية المخروطية, المراجعة المنهجية, التحليل التلوي, Artificial intelligence, Diagnostic modality, Keratoconus, Meta-analysis, Systematic review

## Abstract

**Objectives:**

The challenges in diagnosing keratoconus (KC) have led researchers to explore the use of artificial intelligence (AI) as a diagnostic tool. AI has emerged as a new way to improve the efficiency of KC diagnosis. This study analyzed the use of AI as a diagnostic modality for KC.

**Methods:**

This study used a systematic review and meta-analysis following the 2020 Preferred Reporting Items for Systematic Reviews and Meta-Analyses (PRISMA) guidelines. We searched selected databases using a combination of search terms: “((Artificial Intelligence) OR (Diagnostic Modality)) AND (Keratoconus)” from PubMed, Medline, and ScienceDirect within the last 5 years (2018–2023). Following a systematic review protocol, we selected 11 articles and 6 articles were eligible for final analysis. The relevant data were analyzed with Review Manager 5.4 software and the final output was presented in a forest plot.

**Results:**

This research found neural networks as the most used AI model in diagnosing KC. Neural networks and naïve bayes showed the highest accuracy of AI in diagnosing KC with a sensitivity of 1.00, while random forests were >0.90. All studies in each group have proven high sensitivity and specificity over 0.90.

**Conclusions:**

AI potentially makes a better diagnosis of the KC with its high performance, particularly on sensitivity and specificity, which can help clinicians make medical decisions about an individual patient.

## الملخص

**أهداف البحث:**

دفعت التحديات في تشخيص القرنية المخروطية الباحثين إلى استكشاف استخدام الذكاء الاصطناعي كأداة تشخيصية. لقد ظهر الذكاء الاصطناعي كطريقة جديدة لتحسين كفاءة تشخيص القرنية المخروطية. تهدف هذه الدراسة إلى تحليل استخدام الذكاء الاصطناعي كوسيلة لتشخيص القرنية المخروطية.

**طريقة البحث:**

استخدمت هذه الدراسة مراجعة منهجية وتحليلا تلويا باتباع إرشادات بريزما (عناصر إعداد التقارير المفضلة للمراجعات المنهجية والتحليلات الوصفية) لعام 2020. لقد بحثنا في قواعد بيانات مختارة باستخدام مجموعة من مصطلحات البحث: "((الذكاء الاصطناعي) أو (طريقة التشخيص)) و(القرنية المخروطية)" من "ببميد" و "ميدلاين" و "ساينس دايركت" خلال السنوات الخمس الماضية (2018-2023). بعد بروتوكول المراجعة المنهجية، اخترنا 11 مقالة وكانت 6 مقالات مؤهلة للتحليل النهائي. تم تحليل البيانات ذات الصلة باستخدام برنامج ريفيو مانيجر 5.4.

**النتائج:**

وجد هذا البحث أن الشبكات العصبية هي نموذج الذكاء الاصطناعي الأكثر استخداما في تشخيص القرنية المخروطية. أظهرت الشبكات العصبية أعلى دقة للذكاء الاصطناعي في تشخيص القرنية المخروطية. أثبتت جميع الدراسات في كل مجموعة حساسية ونوعية عالية تزيد عن 0.90.

**الاستنتاجات:**

من المحتمل أن يقوم الذكاء الاصطناعي بتشخيص أفضل للقرنية المخروطية من خلال أدائه العالي، خاصة فيما يتعلق بالحساسية والنوعية، مما يمكن أن يساعد الأطباء على اتخاذ قرارات طبية بشأن المرضى.

## Introduction

Artificial intelligence (AI) has been utilized to enhance diagnostic management and guide treatment. The use of AI as a sophisticated diagnostic modality has not only improved the feature of cornea evaluation in ocular imaging but also has unlocked new pathways for biomechanics, which generates a better understanding of disease pathology.^1^^,^^2^ It has been developed to diagnose keratoconus (KC), but the challenges presented are the need for clinical signs, uniform screening criteria available,^3^ and a low disease prevalence.^2^^,^^4^ The healthy cornea shows a convex aspherical surface and a high refractive index, making it the most vital refractive element of the eyes.^5^ By contrast, KC develops in both eyes with a progressive and asymmetric ectasia corneal disease, characterized by abnormal thinning and bulging of the cornea, resulting in severe vision impairment^6^ and patient dependence on visual aids.^7^ KC primarily starts in adolescence and is more aggressive than in adults.^8^ Thus, for a medical diagnosis, AI can assist in capturing most of the pertinent clinical elements’ complex data, providing prior interpretable outcomes and choosing appropriate treatment strategies.^9^

The challenges in diagnosing KC have prompted scholars to analyze the use of AI in diagnosing the disease.^10^ The latest systematic reviews provided a significant report on KC classification and machine learning algorithm accuracy in detecting KC,^11^^,^^12^ whereas another study focused on imaging modalities in examining deep learning algorithms.^13^ However, these studies did not specifically analyze the accuracy of AI models. Therefore, this study analyzed the accuracy of KC diagnosis based on its classification and the AI model used.

## Materials and Methods

### Source of data and search strategy

A systematic review and meta-analysis were used to examine AI as a diagnostic modality for KC. We followed the 2020 Preferred Reporting Items for Systematic Reviews and Meta-Analysis (PRISMA) guidelines.^14^ The data source was accessible studies in three databases: PubMed, Medline, and ScienceDirect. The search strategy used was a combination of search terms: “((Artificial Intelligence) OR (Diagnostic Modality)) AND (Keratoconus).” We narrowed the search to studies published within the last 5 years (2018–2023). The study protocol can be found on PROSPERO (www.crd.york.ac.uk; Registration No. CRD42023433737).

### Inclusion and exclusion criteria

We included studies that address AI as a diagnostic modality for KC. The criteria for the inclusion of studies were as follow s: (1) published in scholarly journals; (2) published in English; (3) not case reports; and (4) targeted the use of AI to diagnose KC. We excluded reviews, comments, protocols, and reply articles from this review. Moreover, we excluded studies published before 2018.

### Data extraction and quality assessment

After removing duplicate results of records, we reviewed abstracts and titles to identify eligible articles for full-text review. Then quality of the evidence of included articles that met the inclusion criteria was assessed by following Cochrane's recommendation of risk of bias and level of evidence.^15^ The criteria to determine the evidence for the level of risks followed QUADAS-2 (which is designed to assess the quality of primary diagnostic accuracy studies), with the assistance of the RevMan 5.4 tool.

### Statistical analyses

All studies were configured in RevMan 5.4 from Cochrane for Windows. We compared each study's sensitivity and specificity value in the 95 % confidence interval (CI). Then we fit hierarchical summary receiver-operating characteristic curves. Studies on AI models are presented as different shapes, represented by a summary point with a dot surrounded by the 95 % CI.

## Results

### Study selection

**Figure 1:**
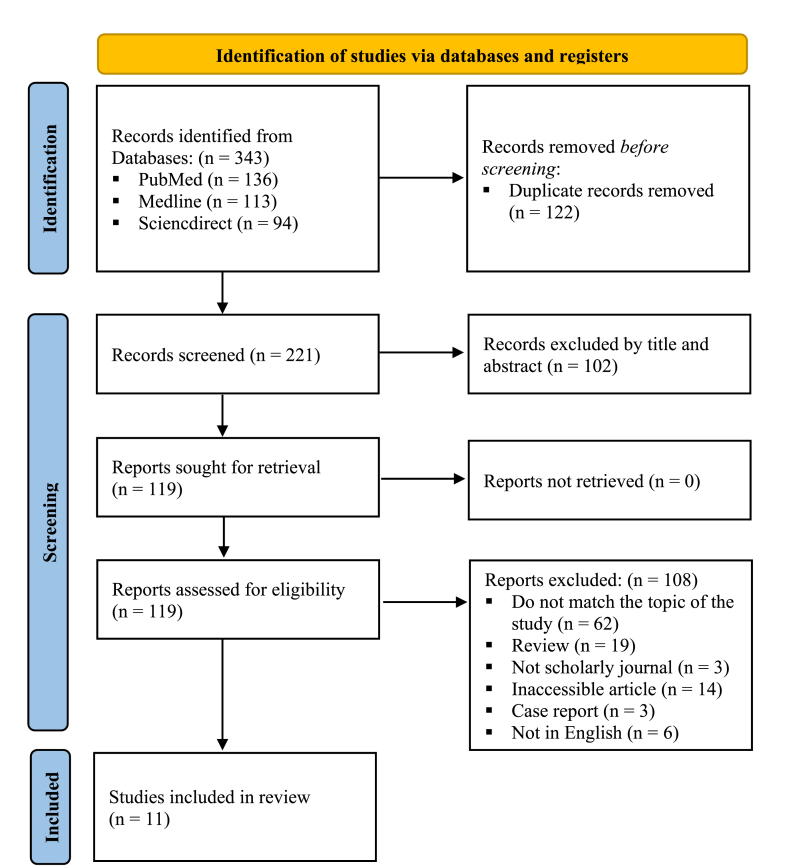
PRISMA 2020 flow diagram.

We identified 343 records in databases and retrieved 221 studies after removing 122 duplication records assisted by Endnote 20 application. We screened the titles and abstracts of 221 studies, excluded 102 records, and retrieved 119. Furthermore, we excluded 108 studies as they failed to meet the inclusion criteria, and retrieved 11 eligible studies for the analyses^9^^,^^16^, ^17^, ^18^, ^19^, ^20^, ^21^, ^22^, ^23^, ^24^, ^25^ (see Figure 1).

The studies were conducted in several countries including China (n = 2),^17^^,^^22^ Australia (n = 2),^19^^,^^20^ Japan (n = 2),^21^^,^^25^ Germany (n = 1),^24^ Belgium (n = 1),^9^ Spain (n = 1),^23^ South Korea (n = 1),^16^ and Taiwan (n = 1).^18^ The earliest studies included were published in 2018, and the latest studies were published in 2021.

### Characteristics of the studies

We reviewed 11 studies on AI and KC and categorized them into three groups: early KC from controls, KC eyes from controls, and KC severity.^9^^,^^16^, ^17^, ^18^, ^19^, ^20^, ^21^, ^22^, ^23^, ^24^, ^25^ To maintain consistency with a previous systematic review, we used the term “early KC” to refer to subclinical or forme fruste KC^12^ (See Table 1).

**Table 1:** Characteristics of the included studies.

1st author/Year	Country	KC Stages	Total Samples/Cases	AI model	Diagnosis Method/parameters	Validation
∗Cao et al.^19^ (2020)	Australia	Early KC	88/49	MM	Pentacam/11	Internal
∗Ahn et al.^16^ (2022)	South Korea	Early KC/KC to control	69/38	FcNN, XGBoost	Pentacam/4	Internal & External
∗Shi et al.^17^ (2020)	China	Early KC/KC to control	121/33	NN	UHR-OCT & Pentacam HR/49	Internal
∗Kuo et al.^18^ (2020)	Taiwan	Early KC/KC to control	354/28	CNN	TMS-4	Internal
Issarti et al.^9^ (2019)	Belgium	Early KC	389/77	FNN	Pentacam/28	Internal & External
Cao et al.^20^ (2021)	Australia	Early KC	267/145	RF	Pentacam/810	Internal & External
Castro-Luna et al.^23^ (2020)	Spain	KC to control	60/30	NB	CSO/16	Internal
Tan et al.^22^ (2022)	China	KC to control	354/143	FNN	Corvis ST/4	External
Kamiya et al.^21^ (2019)	Japan	KC to control	543/304	CNN	AS-OCT, CASIA/6	Internal
Herber et al.^24^ (2021)	Germany	KC to control	126/93	LDA & RF	Pentacam/10	Internal
∗Yousefi et al.^25^ (2018)	Japan	KC severity	3156/-	DBC	CASIA-OCT/420	NA

Three classifications of KC diagnosis: Early KC from control, Clinical KC from control and KC severity. Multiple methods (MMs), Linear Discriminant Analysis (LDA), Neural Network (NN), Feedforward Neural Network (FNN), (Convolutional Neural Network (CNN), Random Forest (RF), Naïve Bayes (NB), Density-based clustering (DBC). (∗) Studies not included in the meta-analysis.

### Quality evidence and risk of bias

**Figure 2:**
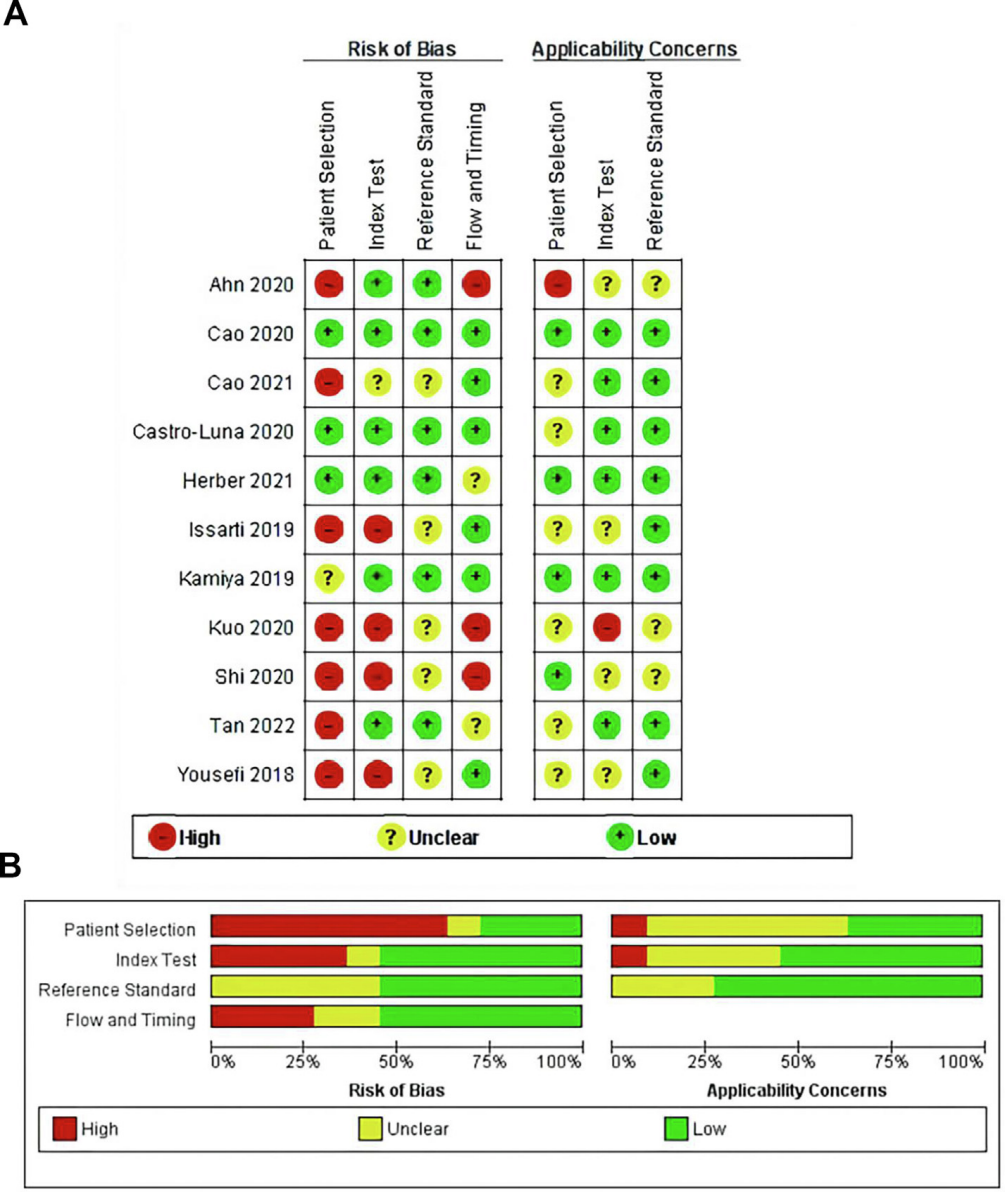
**A**. Risk of bias and applicability concerns summary table. **B.** Risk of bias and applicability concerns graph.

This study used Cochrane's recommendation for QUADAS-2 to assess the quality evidence and risk of bias with the assistance of the RevMan 5.4 tool. Seven of eleven (63.6 %) studies were evaluated with a high risk of bias; the remaining studies were either unclear or had lower risks (Figure 2A, B). High risk of bias studies failed to explain the population (selection of patients), no index test (no data split or cross-validation unreported), or deal with the study flow and time. As no gold standard exists for diagnosing KC, the risk of bias in the models may be influenced by various definitions.

### Use of AI as a diagnostic modality for KC

A meta-analysis was conducted on 6 of 11 studies. Five studies^16^, ^17^, ^18^, ^19^^,^^25^ were omitted due to insufficient data for calculating true positive (TP), false negative (FN), false positive (FP), and true negative (TN). The analysis followed a KC classification, and AI models were used as diagnostic modalities. Two studies in the meta-analysis of KC classification used corneal topography parameters to evaluate AI models in early KC from controls, varying from 28 to 810 parameters.^9^^,^^20^ Those studies had more parameters than those used to identify KC from controls, with only 36 parameters.^21^, ^22^, ^23^, ^24^ Diagnostic reviews encompass the results of sensitivity and specificity from multiple studies.^26^ Thus, we selected and compared either the AI model with the highest sensitivity and specificity from each study or overall sensitivity and specificity scores. We found that the most used imaging machines were Pentacam (including Pentacam HR). Three studies in this meta-analysis used Pentacam data,^9^^,^^20^^,^^24^ one study used CASIA,^21^ and each of the remaining studies used CSO^23^ and Corvis ST.^22^

Most studies (n = 5) used a single machine learning algorithm,^9^^,^^20^, ^21^, ^22^, ^23^ and one study^24^ compared several algorithms. Those studies explored machine learning algorithms and deep learning including naïve bayes (NB), neural networks (NNs), and random forest (RF). NNs (feedforward neural networks [FNN] and convolutional neural networks [CNN]) are the most used AI models for diagnosing KC. Three studies successfully detected KC using a NN system, and two studies employed a FNN to deal with corneal topography for detecting KC.^9^^,^^22^

**Figure 3:**
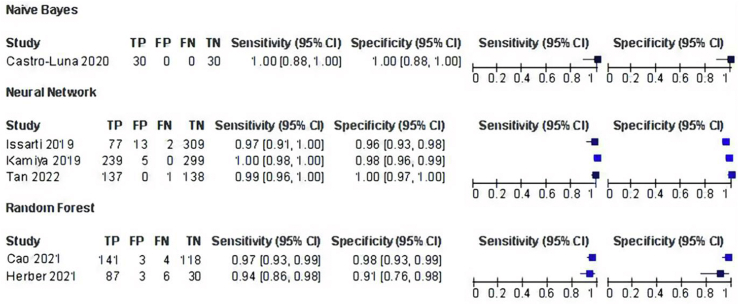
AI performances in KC diagnosis.

The high sensitivity of diagnosing KC was obtained from these two studies in the NN group, which was retrospectively 0.97 (0.91, 1.00) and 0.99 (0.96, 1.00), while the specificity for each study was 0.96 (0.93, 0.98) and 1.00 (0.97, 1.00). One study by Kamiya et al. used deep learning with a CNN algorithm to classify six color-coded maps for detecting KC, resulting in high sensitivity of 1.00 (0.98, 1.00) and specificity of 0.98 (0.96, 0.99)^21^ (see Figure 3).

The second most used AI model to detect KC is RF. Two studies^20^^,^^24^ investigated RF as an AI algorithm to compare KC with normal eyes, which resulted in a sensitivity of 0.97 (0.93, 0.99) and 0.94 (0.86, 0.98) and a specificity of 0.98 (0.93, 0.99) and 0.91 (0.76, 0.98). One study^23^ employed NB to evaluate normal and KC eyes, which had the highest sensitivity and specificity of 1.00 (0.88, 1.00) and 1.00 (0.88, 1.00). Thus, of the three AI models developed, NNs and NB have the highest sensitivity and specificity compared to the RF group.

**Figure 4:**
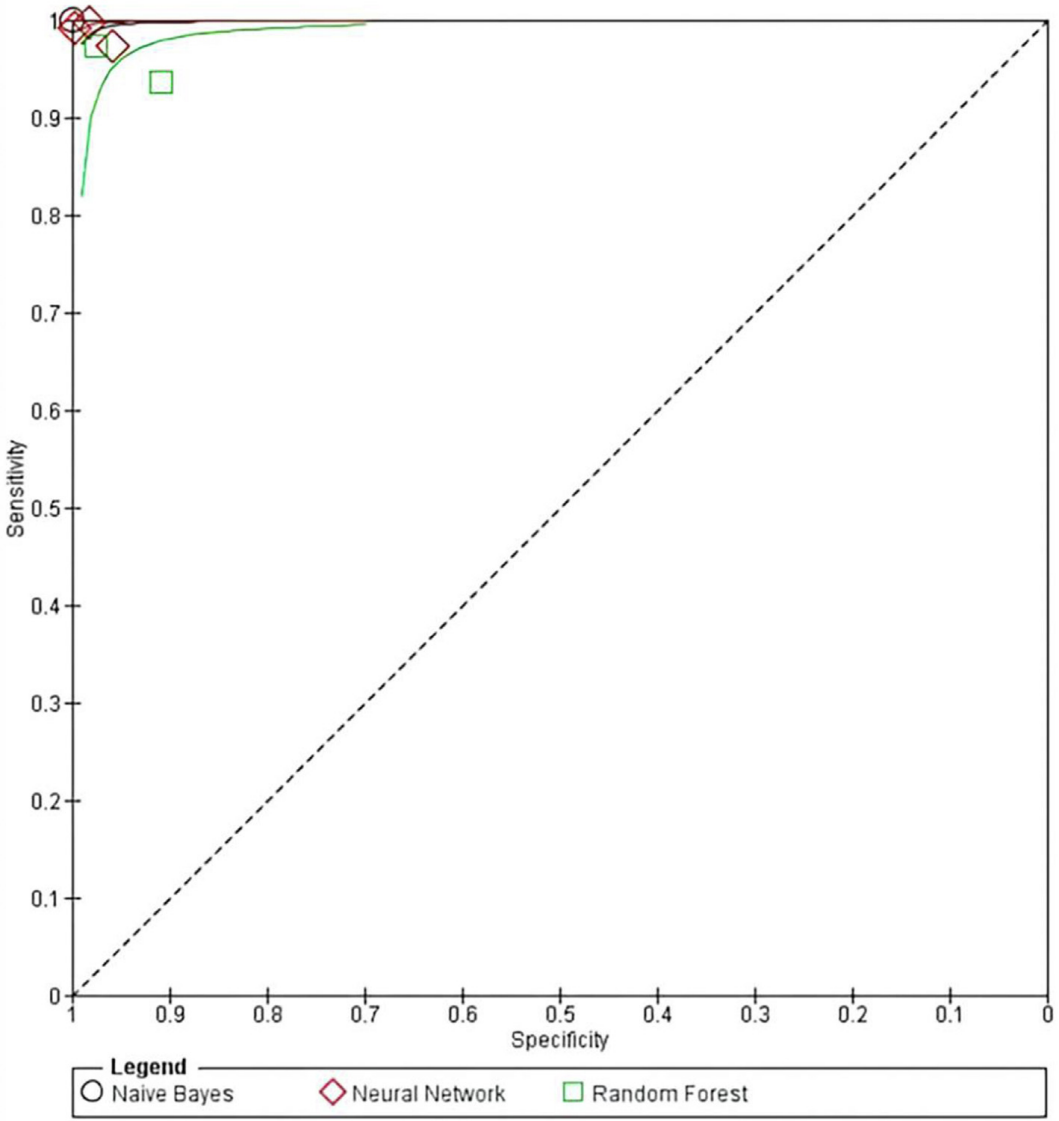
Summary receiver-operating characteristic curve of AI diagnostic performance for KC.

RFs had the lowest sensitivity and specificity. In addition, one study excluded from the meta-analysis developed a classification of KC severity. A study by Yousefi et al. used density-based clustering as an algorithm from CASIA diagnostic labeling to distinguish KC from normal eyes and classify KC severity into several clusters.^25^ Although this unsupervised machine learning identifies distinctive clusters in the data, it showed a high sensitivity of 97.7 % and specificity of 94.1 %. As all studies showed a sensitivity and specificity >0.90, the overall performance of AI models showed significant results. Moreover, from all the studies that used AI, one study on NB, one study on RFs, and two studies on NNs investigated the KC diagnosis compared to the control group, while one study on NN and one study on RF included early KC versus the control group. The summary of the performance of AI-assisted diagnosis can be seen in Figure 4.

## Discussion

The meta-analysis on KC and AI demonstrated that AI can be reliable for differentiating KC eyes from control eyes by utilizing data from various corneal imaging devices. However, as AI models have been developed for various imaging systems, they are not literally exchangeable due to different selected inputs and expectations. NN, for instance, has been proven to have greater sensitivity in diagnosing KC. Nevertheless, the study conducted by Cao et al., which used multilayer perceptron NN, showed that the algorithm has significantly low sensitivity (0.80) and specificity (0.20) compared to other AI models.^19^ This result differs from a study conducted by Valdés-Mas et al., which found that multilayer perceptron showed the best results from all models they proposed.^27^ Meanwhile, using similar Pentacam data, Tan et al. discovered that the performance of the FNN model with sensitivity and specificity was higher than 0.90.^22^

Our research discovered that NN is the most frequently used AI model for diagnosing KC with high accuracy^9^^,^^21^^,^^22^ Lavric et al. argued that the accuracy of the NN lies in the simultaneous use of the topographic parameters to both eyes, which enhances its discriminative ability.^28^ The more cases used as input, the more this AI makes the output more predictable and accurate, as a learning process can enhance it, and the program keeps learning that generates changes in its behavior.^29^

NB has a sensitivity and specificity of 1.00 even in the presence of very significant noise. It is a probabilistic classifier that is well suited for high dimensional datasets. The results of this review are in line with the study by Luis et al., which demonstrated that the best performing classification algorithm was NB with an accuracy of 0.9401 (95 % Cl: 0.93 to 0.94) compared to other algorithms for KC detection.^23^

RF was found to be the second most commonly used model for detecting KC, with a ensitivity and specificity >0.90.^20^^,^^24^ The advantage of this model is that the forest is relatively stable, even though the data and performances of individual trees change due to the combination of many trees.^30^^,^^31^ Lopes et al. assessed the accuracy of this model compared with other models, and found that RF had the best performance in detecting subclinical eyes, with a sensitivity of 85.2 %.^31^ A study detecting KC severity also discovered that RF has the highest classification performance compared with other parameters with an accuracy of 98 %.^32^ AI can potentially make better diagnoses of the KC with their high performances, particularly on sensitivity and specificity. The main objective of AI in healthcare services is to support clinicians in making medical decisions concerning patients. The diagnosis of KC should distinguish between the early and later stages of KC in order to provide the appropriate treatment. Finally, given that the problem with KC diagnosis is selecting the input to be analyzed that affects the high or low performance of diagnosis, further studies need to select the most suitable inputs for each AI classifier to maximize the AI performance.

## Conclusion

This study presents the prospect of using AI models to diagnose KC. Pentacam is the most commonly used machine learning method, whereas the NN is the AI algorithm frequently used to help find a more accurate diagnosis. The AI models with the highest sensitivity and specificity detection are NNs and NB with a sensitivity reaching 1.00. Due to the complexity of quantifying the pooled sensitivity and specificity for more than one AI model in each group, this study was limited to conducting a meta-analysis on one AI model for each study. Thus, additional studies are needed on more AI models as diagnostic modalities for KC with sufficient data on TP, FN, FP, and TN. As the challenges in conducting a diagnosis are the selection of inputs, it is recommended that the clinician determine the most appropriate inputs for each AI model. Despite the limitations, this study significantly contributes to providing evidence of AI's accuracy as a diagnostic modality for KC.

## Source of funding

This research did not receive any specific grant from funding agencies in the public, commercial, or not-for-profit sectors.

## Conflict of interest

The authors have no conflict of interest to declare.

## Ethical approval

There are no ethical or financial issues or animal experiments related to this research.

## Authors contributions

AA contributed to the conception of the research, and conducted the systematic review and meta-analysis; FS and PMA contributed significantly to the literature search, quality assessment, and improving the article for language and style; DD revised the manuscript and approved the final version. All authors have critically reviewed and approved the final draft and are responsible for the content and similarity index of the manuscript.

## Acknowledgment

The authors would like to thank the Department of Ophthalmology, Undaan Eye Hospital and Faculty of Medicine, 10.13039/501100016274Universitas Sriwijaya for the research support.
